# Notfallmedizinische Aspekte bei Laufveranstaltungen

**DOI:** 10.1007/s10049-021-00959-w

**Published:** 2021-12-02

**Authors:** Simon-Richard Finke, Christoph Jänig, Andreas Deschler, Jan Hanske, Holger Herff, Jochen Hinkelbein, Bernd W. Böttiger, W. Schmidbauer, Daniel C. Schroeder

**Affiliations:** 1 Klinik für Anästhesiologie und Operative Intensivmedizin, Universitätsklinikum Köln, Kerpener Str. 62, 50937 Köln, Deutschland; 2 Klinik X – Anästhesie, Intensivmedizin und Notfallmedizin, Bundeswehrzentralkrankenhaus Koblenz, Rübenacher Str. 170, 56072 Koblenz, Deutschland; 3 Flugbereitschaft des Bundesministeriums der Verteidigung, Flughafenstr. 1, 51147 Köln, Deutschland

**Keywords:** Marathon, Laufen, Plötzlicher Herztod, Sportverletzungen, Wiederbelebung, Marathon, Jogging, Sudden cardiac death, Athletic injuries, Cardiopulmonary resuscitation

## Abstract

**Hintergrund:**

Die Anzahl an kurz- und langstreckigen Laufveranstaltungen in Deutschland nimmt zu. Laufen als Breitensport wird von einer Vielzahl an Personen unterschiedlicher Altersklassen, Risikogruppen und Professionalisierungsgrade betrieben, woraus ein breites Spektrum medizinischer Notfälle resultiert.

**Ziel der Arbeit:**

Der vorliegende Beitrag erläutert die Inzidenz, Pathophysiologie und Therapie relevanter Notfallbilder bei Laufveranstaltungen. Ziel ist die Optimierung der Arbeitsabläufe des Rettungsdienstpersonals.

**Material und Methoden:**

Es erfolgte eine Literaturrecherche in PubMed.

**Ergebnisse:**

Anstrengungsassoziierte Muskelkrämpfe, gastrointestinale Symptome, Kollaps, Kompartmentsyndrom und Tendinopathien sind häufige Erscheinungsbilder und resultieren meist aus akuter oder chronischer Überanstrengung. Der Kreislaufstillstand bzw. plötzliche Herztod ist ein seltenes Ereignis bei Laufveranstaltungen. Bewusstseinsstörungen und generalisierte Krampfanfälle sind schwerwiegende Komplikationen. Disseminierte intravasale Koagulopathie, belastungsinduzierte Hyponatriämie, Hitzschlag, Rhabdomyolyse und thromboembolische Ereignisse sind mit hoher Morbidität und Mortalität verbunden. Substanzen zur Erhöhung der Schmerzschwelle und leistungssteigernde Substanzen sind unter Läufern stark verbreitet und gehen mit einer hohen Rate an Nebenwirkungen einher.

**Diskussion:**

Unspezifische Symptome wie Erbrechen, Fieber, Kollaps, Muskelschmerzen, Übelkeit, und Schwäche sind die führenden Symptome bei Laufveranstaltungen. Eine sorgfältige Anamnese ist wegweisend für eine zielgerichtete klinische Therapie. Präklinisch steht eine Symptomkontrolle im Mittelpunkt. Das Flüssigkeitsmanagement stellt eine besondere Herausforderung dar.

## Abstract (en)

**Background:**

The number of short- and long-distance running events in Germany is increasing. Running as a popular sport is practiced by a large number of people of different ages, risk groups, and degrees of professionalism, which results in a wide range of medical emergencies.

**Objective:**

The present article elucidates incidence, pathophysiology and therapy of relevant emergencies during running events. Aim was the optimization of work processes of emergency personnel.

**Materials and methods:**

A literature search was conducted in PubMed.

**Results:**

Exercise-associated muscle cramps, gastrointestinal symptoms, collapse, compartment syndrome, and tendinopathy are common clinical manifestations. Cardiac arrest and sudden cardiac death are rare events. Consciousness and seizures are major complications. Disseminated intravascular coagulation, exercise-associated hyponatremia, heat stroke, rhabdomyolysis, and thromboembolism are associated with high morbidity and mortality. Substances increasing pain resilience as well as performance-enhancing substances are popular among amateur and professional runners and are associated with a high incidence of side effects.

**Conclusion:**

General symptoms including vomiting, fever, collapse, muscle-pain, nausea and weakness are the leading symptoms during running events. A careful anamnesis is important for targeted clinical therapy. Symptom control is the main task. Fluid management the most challenging task for healthcare providers in the prehospital setting.

## Infobox 1 Empfehlungen zum Umgang mit dehydrierten Patienten. (Mod. nach [1] und [2])

- Symptomkontrolle/Sicherung der Vitalfunktionen
- Denke an eine Hyponatriämie als Differenzialdiagnose.
- Achte bei der Untersuchung (ABCDE-Schema) auf eine gewissenhafte Beurteilung des neurologischen Status.
- Messe den Blutzuckerspiegel.
- Erwäge Point-of-Care-Diagnostik.
- Vermeide unkontrollierte Flüssigkeitszufuhr (restriktives Flüssigkeitsmanagement).
- Verwende 0,9 %ige NaCl-Lösung oder balancierte Vollelektrolytlösungen zur intravenösen Substitution.
- Überwache Patienten mit milden Symptomen mindestens 60 min. Stelle eine großzügige Indikation zur Messung des Serumnatriumspiegels, was meist mit der Einweisung ins Krankenhaus verbunden ist.
- Substituiere 10–20 ml/kg einer geeigneten Infusionslösung bei klinisch instabilen Patienten (z. B. anhaltende Hypotonie).
- Weise Patienten mit nicht eindeutig einzuordnender Symptomatik und schweren Verläufen großzügig ins Krankenhaus ein.

## Hinführung zum Thema

Das Angebot an kurz- und langstreckigen Laufveranstaltungen ist innerhalb der letzten 10 Jahre stark angestiegen. Einzelne Halbmarathonveranstaltungen verzeichnen bis zu 28.000 Teilnehmer. Im Jahr 2019 wurden in Deutschland 238 Marathonwettkämpfe durchgeführt, bei denen über 110.000 Teilnehmer das Ziel erreichten [3], was schätzungsweise 92 % der Starter entspricht [4].

Laufen als Breitensport wird von einer Vielzahl an Personen unterschiedlicher Altersklassen, Risikogruppen und Professionalisierungsgrade betrieben. Aus dieser inhomogenen Population resultiert ein breites Spektrum medizinischer Notfälle, mit denen der Rettungsdienst auf Laufveranstaltungen konfrontiert wird. Zudem beeinflussen Wetterbedingungen, Unterhaltungs- und Rahmenprogramme sowie hohe Zuschaueraufkommen die Einsatzbilder.

Der vorliegende Beitrag erläutert pathophysiologische Hintergründe und Therapieansätze präklinisch relevanter Notfallbilder bei Laufveranstaltungen. Ziel der Arbeit ist die Optimierung der Arbeitsabläufe von nichtärztlichem und ärztlichem Rettungsdienstpersonal und eine Vertiefung der Expertise der zu erwartenden Erkrankungen und Verletzungsmuster.

## Verletzungs- und Erkrankungsbilder

### Weichteilsystem

#### Anstrengungsassoziierte Muskelkrämpfe

Als anstrengungsassoziierter Muskelkrampf (AAMK) wird eine schmerzhafte, spastische, unwillkürliche Skelettmuskelkontraktion bezeichnet, die während bzw. unmittelbar nach Belastung auftritt [5, 6]. Als Ursache werden muskuläre Überlastung [5], Elektrolytverschiebungen [6] und eine geringe Trinkmenge diskutiert [6, 7]. Eine Häufung des AAMK zeigt sich bei älteren und untrainierten Sportlern sowie auf dem Boden einer Gelenkfehlstellung [5, 6]. In Muskelgruppen mit starker Kontraktionsfähigkeit und hoher Beanspruchung treten ebenfalls vermehrt AAMK auf [6]. Charakteristisch sind anhaltende, schmerzhafte Muskelkontraktion und Bewegungsunfähigkeit der beanspruchten Muskelgruppe während oder unmittelbar nach Belastung [5, 6]. Therapeutisch werden eine vorsichtige Dehnung des Muskels und die orale Rehydrierung empfohlen [5, 6].

#### Tendinopathien

Aus Überbeanspruchung der Sehnen resultiert eine progressive Degeneration von Kollagenfasern [8, 9]. Aufgrund fehlender Reparaturmaßnahmen kommt es zu Schmerz bzw. Funktionsverlust (Tendinopathie). Dieser muss nicht zwangsläufig mit einem strukturellen Schaden – z. B. einem Riss – einhergehen [9]. Eine Achillessehnentendinopathie zeigt sich bei Kurzstreckenläufern gehäuft [10]. Die Plantarfasziitis geht mit einer Degeneration der Plantarfaszie einher und ist der häufigste Grund für Schmerzen der Ferse ([11]; Tab. 1). Klinisch zeigt sich ein häufig vorbekannter Ruhe- oder Belastungsschmerz [9–11]. Die präklinische Therapie besteht in Schonung der betroffenen Extremität und Analgesie [12, 13].

**Table 1:** 

Weichteilsystem		Knöchernes System	
Verletzung	Inzidenz (%)	Verletzung	Inzidenz (%)
Patellasehnentendinopathie	5,5–22,7	Mediales tibiales Stresssyndrom	13,6–20,0
Achillessehnentendinopathie	9,1–10,9	Verstauchung des Sprunggelenks	10,9–15,0
Zerrung der Oberschenkelmuskulatur	10,9	Stressfraktur der Tibia	9,1
Muskelfaserriss	10	Verstauchung des Kniegelenks	5
Plantarfasziitis	4,5–7,3	Weitere Frakturen	4,5–5,0

##### Merke

Verletzungen des Weichteilapparats gehen häufig mit unspezifischen Symptomen einher [10]. Die präklinische Diagnose ist schwierig. Die Beendigung des Wettkampfs kann folgenreiche Schäden verhindern.

#### Rhabdomyolyse

Eine Rhabdomyolyse kann durch Traumata, Medikamente und Noxen sowie eine ausgeprägte thermische Belastung (Hitzschlag) ausgelöst werden [15]. Der Rhabdomyolyse liegt eine Schädigung der Zellmembran der Skelettmuskelzelle (Sarkolemm) oder ein Zusammenbruch der Energieversorgung mit Dysfunktion der Na^+^/K^+^- und Ca^++^-ATPase zugrunde. Es resultiert eine erhöhte Permeabilität für Na^+^ nach intrazellulär mit konsekutivem Anstieg der intrazellulären Ca^++^-Konzentration. Nach Aktivierung Ca^++^-abhängiger Proteasen und Phospholipasen kommt es zur Schädigung von Zellstrukturen mit Freisetzung von Proteinen (Myoglobin, Kreatininkinase, Laktatdehydrogenase) und Metaboliten (K^+^, Phosphat; [15]). Erhöhte Serummyoglobinspiegel führen zur mechanischen Verlegung der Nierentubuli und begünstigen ein akutes Nierenversagen [15, 16]. Zudem können freigesetzte Proteine und Elektrolyte zu Herzrhythmusstörungen, Kreislaufstillstand, einer disseminierten intravasalen Gerinnung oder einer Ödematisierung der Muskulatur mit Kompartmentsyndrom führen [15]. Unspezifische Anfangssymptome wie Schmerzen, Schwäche und Bewegungseinschränkungen weisen auf eine Schädigung der Skelettmuskulatur hin, deren Ausmaß nicht unterschätzt werden darf. Präklinisch sollte eine symptomatische Therapie (Analgesie, Kühlung) erwogen werden [15]. Bei unklarer Diagnose sollte eine großzügige Einweisung ins Krankenhaus erfolgen.

##### Merke

Hinweise auf eine größere Schädigung der Skelettmuskulatur benötigen eine serologische Abklärung zum Ausschluss von Nierenschäden und Elektrolytstörungen im Krankenhaus.

### Knöchernes System

#### Frakturen

Chronische Überbeanspruchung des Knochens führt zunächst zu einer Verschiebung des Gleichgewichts im Knochenstoffwechsel mit vermehrtem Knochenabbau. Bei fortgesetzter Belastung unterhalb der Frakturschwelle entstehen Mikrofrakturen, die bei ausbleibenden Reparaturmechanismen in einer (Stress‑)Fraktur münden ([17]; Tab. 1). Betroffen sind vorwiegend Tibia, Fibula, Mittelfußknochen sowie der Beckenkamm [18]. Klinisch zeigt sich ein vorbestehender, zunehmender Belastungsschmerz, der durch Palpation lokalisierbar ist und von unspezifischen Symptomen wie Rötung und Schwellung begleitet wird [18]. Schonung, Schienung und Analgesie stellen präklinische Therapieansätze dar [18]. Die Fortsetzung des Wettkampfs kann nicht empfohlen werden. Darüber hinaus kann es bei Laufveranstaltungen zu Frakturen und Gelenkverletzungen (Tab. 2 und 3) infolge akuter Kraft- bzw. Gewalteinwirkung kommen. Erschöpfung, Unachtsamkeit oder vorbestehende gesundheitliche Einschränkungen können einen Sturz bewirken und müssen anamnestisch abgeklärt werden.

**Table 2:** 

	Prellung (Kontusion)	Zerrung/Dehnung (Distorsion)	Verrenkung (Luxation)
Pathomechanismus	Direkte stumpfe Gewalteinwirkung ohne Verschiebung der Gelenkflächen gegeneinander	Verschiebung der gelenkbildenden Teile mit Überschreitung des physiologischen Bewegungsumfangs und anschließender Rückkehr in die Ausgangsposition	Dauerhafte Verschiebung der gelenkbildenden Elemente (vollständig/unvollständig)
Geschädigte Struktur	Schäden am Gelenkknorpel	Überdehnung der Gelenkkapsel	Schädigungen des Kapselbandapparats
	Einblutung in den Gelenkraum	Schäden/Einblutung der Gelenkkapsel möglich	Knorpel‑, Knochenverletzungen

**Table 3:** 

Akutes Koronarsyndrom (ACS)	Atemnot, akuter Brustschmerz, STEMI, NSTEMI, Arrhythmien, Leistungseinschränkung, Übelkeit, Erbrechen, Schwitzen, Synkope, Lungenödem, Zyanose [19]	Leitlinienbasierte Therapie [19]
Belastungsinduzierte Hyponatriämie	Lungenödem, neurologische Symptomatik (siehe akutes Hirnödem), präklinisch Ausschlussdiagnose [20, 21]	Zurückhaltende Substitution mit Vollelektrolytlösung [19]
Frakturen	Gelenkfehlstellung, Krepitation, Ruhe- und Belastungsschmerz [17]	Schienung, Analgesie, ggf. Reposition [17]
Akutes Hirnödem	Milde Symptomatik: Kopfschmerzen, Schwindel, Übelkeit, Erbrechen, retrograde Amnesie, Verwirrtheit; schwere Symptomatik: Bewusstseins- und Atemstörungen, Cushing-Reflex, Lähmungen, neurogenes Lungenödem, Pupillendifferenz, Beuge- und Strecksynergismen, Krampfanfall [20, 22]	Symptomatisch, Sicherung der Vitalfunktionen
Hypoglykämie	Übelkeit, Erbrechen, Vigilanzminderung, Verwirrtheit, Unruhe, Schwäche, Krampfanfall [23]	Sicherung der Vitalfunktionen, intravenöse Zufuhr von Glukose [23]
Kreislaufstillstand	Atemstillstand, Bewusstlosigkeit, fehlende Lebenszeichen [24, 25]	Herz-Lungen-Wiederbelebung nach Leitlinien [25]
Lungenarterienembolie	Plötzliche Luftnot, Tachykardie, Brustschmerz, Husten, ggf. blutiger Auswurf, hämodynamische Instabilität, rechtsventrikuläre Belastung, Trikuspidalinsuffizienz [26]	Sofortige therapeutische Antikoagulation, Katecholamine, moderate Flüssigkeitsgabe, O_2_-Applikation, umgehende Einweisung mit Reperfusionsmöglichkeit, instabile Patienten: Lyse erwägen [26]
Orthostatische Dysregulation	Hypotension mit (hyperadrenerge Form) und ohne Tachykardie (hypoadrenerge Form) bzw. mit Bradykardie (neurokardiogene Form), Bewusstlosigkeit [22, 27]	Konservative Maßnahmen: Sympathomimetika, orale Flüssigkeitszufuhr, Trendelenburg-Lagerung, „begleitetes Gehen“ zur Aktivierung der Muskelpumpe (cave: Hypertension im Liegen; [28])
Schienbeinkantensyndrom	Belastungsschmerz im distalen Bereich der posteromedialen Tibiakante [29]	Schonung, Analgesie [29]
Schwerwiegender Kollaps	Tachykardie, Hyperthermie, metabolische Azidose, Hypernatriämie [30]	Symptomatisch, Stabilisierung des Kreislaufs [30]
Tiefe Beinvenenthrombose	Schmerzen, Krämpfe, Ödematisierung, Überwärmung, Zyanose, livide Verfärbung, Ödem [26]	Symptomatische Therapie, klinischer Ausschluss Lungenarterienembolie [26]
Traumatische Blutung	Tachykardie, Hypotension, Kaltschweißigkeit, Rekapillarisierungszeit > 2 s [31]	Blutstillung (Kompression, Tourniquet, Beckenschlinge), Tranexamsäure erwägen [31]
Herzrhythmusstörungen (HRST)	Tachykarde HRST: > 100/min; Bradykarde HRST: < 60/min; Palpitationen, Synkope, Übelkeit, Erbrechen, ACS, Herzinsuffizienz [25]	Symptomatisch, Stabilisierung des Kreislaufs, siehe ILCOR-Leitlinien [25]
Prellung (Kontusion)	Druckschmerz, Weichteilschwellung, Bewegungseinschränkung [12, 13]	Analgesie, abschwellende Maßnahmen, Ruhigstellung [12, 13]
Zerrung/Dehnung (Distorsion)	Schmerz über der betr. Bandstruktur, Weichteilschwellung, Bewegungseinschränkung, Dehnungsschmerz [12, 13]	Analgesie, abschwellende Maßnahmen, Ruhigstellung [12, 13]
Verrenkung (Luxation)	Gelenkfehlstellung, leere Gelenkpfanne, Schmerz, Schwellung, Bewegungseinschränkung [12, 13]	Reposition bei Verlust der Durchblutung, Sensibilität erwägen, Klinikeinweisung, Analgesie, Ruhigstellung [12, 13]

*STEMI* ST-Strecken-Elevations-Myokardinfarkt, *NSTEMI* Nicht-ST-Strecken-Elevations-Myokardinfarkt, *ILCOR* International Liaison Committee on Resuscitation

#### Kompartmentsyndrom

Traumata und wiederkehrende hohe körperliche Anstrengung führen zur Volumenzunahme in den Muskellogen. Die Volumenzunahme verursacht einen akuten oder chronischen interstitiellen Druckanstieg mit Beeinträchtigung der Gewebsperfusion. Entstandenes ischämisches Muskelgewebe führt zur Ausbildung eines Ödems, sodass Druckanstieg und Minderperfusion verstärkt werden. Im Rahmen von Laufveranstaltungen ist vor allem die vordere Schienbeinmuskulatur betroffen [29]. Die Symptome sind zunächst unspezifisch (Krämpfe, Muskelschwäche). Später fällt eine klinische Härtung des Muskels bei Bewegung mit begleitendem Ruheschmerz, Hypästhesie und Minderdurchblutung auf [29, 32]. Neben Schonung und Analgesie müssen Durchblutung, Motorik, Sensibilität (DMS) überprüft werden [29, 32]. Das fortgeschrittene Kompartmentsyndrom erfordert eine zeitnahe chirurgische Intervention in der Klinik.

##### Merke

Die präklinische Diagnose des Kompartmentsyndroms ist entscheidend für die Prognose des Patienten [29].

### Herz-Kreislauf-System

#### Plötzlicher Herztod/Kreislaufstillstand

Die Inzidenz des Kreislaufstillstands bei Laufveranstaltungen liegt zwischen 1:15.000 und 1:150.000 [33–35]. Die Datenlage zu Überlebensraten ist schwach. Kim et al. berichten, dass 71 % der Teilnehmer eines Halbmarathons bzw. Marathons nach Kreislaufstillstand versterben, was einer Inzidenz von einem Todesfall pro 259.000 Teilnehmer entspricht [34]. Webner et al. zeigten in einer retrospektiven Analyse eine Inzidenz von einem Todesfall pro 171.005 Teilnehmer [36]. Der Kreislaufstillstand betrifft vor allem Männer (93 %) mit einem Durchschnittsalter von 45 bis 50 Jahren, ist in 65–70 % mit einer koronaren Herzkrankheit (KHK) vergesellschaftet und tritt gehäuft auf den letzten 5 km eines Marathons auf [34, 36]. Eine vorbestehende hypertrophe Kardiomyopathie geht mit einer hohen Mortalität bei Laufveranstaltungen einher [34] und ist die Hauptursache für Kreislaufstillstände bei jüngeren Sportlern [37]. Hyponatriämie und Hitzschlag sind seltene Gründe für einen Kreislaufstillstand [34]. Ein früher Beginn der Wiederbelebungsmaßnahmen und initiales Kammerflimmern sind Prädiktoren für die Wiederherstellung eines Kreislaufs [34].

##### Merke

Die Sicherstellung von Sanitäts- und Rettungsdienstpersonal sowie die Möglichkeit der (Früh‑)Defibrillation auf den letzten 10 km eines Marathons können die Überlebensrate steigern [34].

Pathophysiologisch existieren verschiedene Erklärungsmodelle für die Entstehung eines Kreislaufstillstands bei Laufveranstaltungen. Durch Rhabdomyolyse und Flüssigkeitsverlust kommt es zu Elektrolytverschiebungen, woraus hämodynamisch relevante kardiale Arrhythmien resultieren (Tab. 3; [15, 38]). Zudem begünstigt infolge körperlicher Aktivität freigesetztes Interleukin‑6 das Syndrom der inadäquaten Ausschüttung von antidiuretischem Hormon, was wiederum die Natriumausscheidung fördert und eine Hyponatriämie begünstigt [20, 39].

Darüber hinaus trägt eine belastungsinduzierte Zytokinausschüttung zur Ruptur instabiler Plaques in den Herzkranzgefäßen bei, was durch steigende Troponinwerte bereits bei asymptomatischen Läufern abgebildet wird [40]. Eine relative Dehydratation sowie eine belastungsbedingte prokoagulatorische Blutgerinnung erhöhen das Risiko für kardiale Ereignisse im Vergleich zur Normalbevölkerung nach einem Langstreckenlauf temporär [39]. Die novellierten Wiederbelebungsleitlinien empfehlen, dass der Kollaps eines Sportlers eine „unmittelbare Reaktion … des medizinischen Teams“ auslösen sollte. In diesem Rahmen müssen im Vorfeld Zugangswege zum Patienten für den Rettungsdienst geschaffen werden und bekannt sein. Die Möglichkeit zur (Laien‑)Defibrillation muss jederzeit bestehen. Die Laienausbildung aller Sportler in Wiederbelebungsmaßnahmen soll gesteigert werden. Bei erhöhtem Zuschaueraufkommen kann ein Transport in eine geeignete Umgebung nach drei aufeinanderfolgenden Defibrillationsversuchen unter Fortführung effektiver Thoraxkompressionen erwogen werden. Die Wiederbelebungsmaßnahmen sollen dem allgemeinen Wiederbelebungsalgorithmus folgen [41].

#### Kollaps

Der Kollaps in Folge einer klassischen orthostatischen Dysregulation ist mit 59 % der medizinischen Kontakte die häufigste Komplikation bei Langstreckenläufen. Ein Großteil der Patienten benötigt keine medizinische Betreuung. Wird diese benötigt, spricht man von einem „schwerwiegenden Kollaps“ [30], der mit Tachykardie, Hyperthermie, metabolischer Azidose und Hypernatriämie als Zeichen der temporären Dehydratation einhergeht. Präklinisch stehen die ausführliche Anamnese und die Sicherung der Vitalfunktionen nach dem ABCDE-Schema im Mittelpunkt.

### Wasser‑, Elektrolythaushalt

#### Belastungsinduzierte Hyponatriämie

Ein Serumnatriumspiegel von < 135 mmol/l wird als Hyponatriämie definiert und tritt bei 13 % aller Langstreckenläufer auf [22]. Eine belastungsinduzierte Hyponatriämie verläuft meist asymptomatisch und ist spontan reversibel [20, 27]. Folgende Mechanismen tragen zur Entstehung bei:

- Vermehrter oraler Konsum von hypotoner Flüssigkeit (hypervolämische Hyponatriämie; [20, 30])
- Belastungsinduzierte Hyperthermie mit Abgabe von Wärmeenergie über Verdunstung (Evaporation), in deren Folge es zu Flüssigkeitsverlust mit Dehydratation und Hyponatriämie (hypovolämische Hyponatriämie) kommt [20, 30]

Von einer schweren (belastungsinduzierten) Hyponatriämie spricht man ab einem Serumnatriumspiegel von < 125 mmol/l [42]. Aufgrund einer entstehenden intrazellulären Flüssigkeitszunahme mit Ödematisierung der Zellen besteht die Gefahr eines akuten Hirnödems mit Anstieg des intrakraniellen Drucks [20]. In diesem Rahmen kommt es zunächst zu unspezifischen Symptomen wie Übelkeit, Erbrechen, Ataxie, und Orientierungsstörungen. Quantitative Bewusstseinsstörungen, generalisierte Krampfanfälle und Lungenödem mit Atemnot sind Symptome der schweren Hyponatriämie [1, 42].

Die klinische Diagnose der belastungsinduzierten Hyponatriämie ist äußerst schwierig, da sie leicht mit einem Hitzschlag oder einer Dehydratation verwechselt werden kann [2, 43]. Eine exakte präklinische Differenzierung des Schweregrads ist unmöglich. Nur durch Bestimmung des Serumnatriumspiegels kann die Diagnose der belastungsinduzierten Hyponatriämie gestellt werden [2]. Die gewissenhafte klinische Untersuchung nach dem ABCDE-Schema und die sorgfältige Erhebung des neurologischen Status haben daher einen hohen Stellenwert in der präklinischen Einschätzung von Patienten bei Laufveranstaltungen. Differenzialdiagnostisch sollte eine Hypoglykämie präklinisch ausgeschlossen werden [2].

##### Merke

Bei der Betreuung einer großen Anzahl an Patienten sollte insbesondere auf das Auftreten von Bewusstseinsstörungen und generalisierten Krampfanfällen geachtet werden, die Ausdruck fortgeschrittener Elektrolytstörungen sind und auch mit Latenz nach Beendigung der Belastung auftreten können [2].

Die Symptomkontrolle steht im Mittelpunkt therapeutischer Ansätze bei Laufveranstaltungen (Tab. 3; [2, 44]). Nach Beendigung der Belastung kann es zur weiteren Absorption von Flüssigkeit aus dem Gastrointestinaltrakt kommen, was in der Zunahme der Symptome münden kann [2]. Daher sollte bereits bei Verdacht auf eine milde Hypotonie eine lückenlose Überwachung und zeitnahe Einweisung ins Krankenhaus erfolgen. Patienten mit Verdacht auf eine schwere Hyponatriämie bedürfen einer schnellen intensivmedizinischen Therapie.

Das Flüssigkeitsmanagement stellt eine besondere Herausforderung unter präklinischen Bedingungen dar. Eine rasche Volumentherapie geht mit der Gefahr des weiteren Abfalls des Serumnatriumspiegels einher. Grundlage für eine adäquate Flüssigkeitssubstitution (Rehydratation) ist daher die Kenntnis des Serumelektrolytspiegels, was präklinisch nur erschwert möglich ist [2].

Bei Patienten mit milden Symptomen sollte die Flüssigkeitszufuhr oral erfolgen [44, 45]. Die intravenöse Flüssigkeitszufuhr sollte kritisch kranken Patienten mit einer schweren Dehydratation (Verlust von > 7 % des Körpergewichts, Hypotonie) oder eingeschränkter oraler Aufnahme (Übelkeit, Erbrechen) vorbehalten bleiben und unter engmaschiger klinischer Überwachung durchgeführt werden [44, 45]. Zur intravenösen Substitution können 0,9 % NaCl-Lösung oder balancierte Vollelektrolytlösungen verwendet werden ([1]; Infobox 1).

##### Merke

Schnelle Marathonläufer nehmen während des Laufs geringere Mengen Flüssigkeit zu sich, während bei langsameren Läufern ein „Übertrinken“ beobachtet werden konnte. Schnelle Läufer sind eher durch Dehydratation, langsame Läufer eher durch eine Hyponatriämie gefährdet [45].

#### Hitzschlag

**Figure 1:**
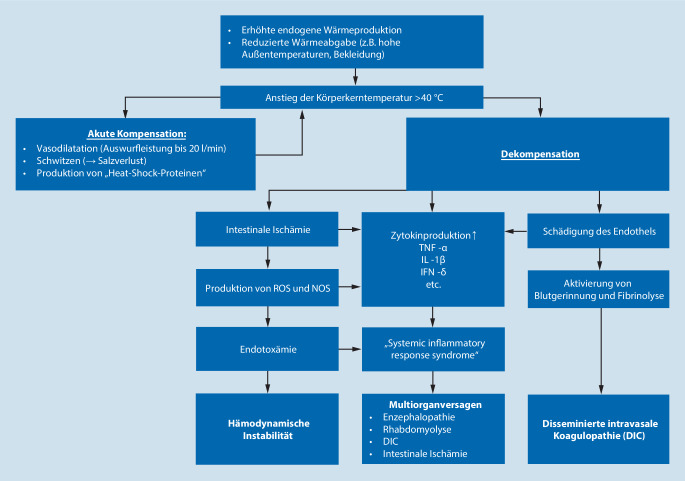

Dem Hitzschlag liegt eine Imbalance zwischen Wärmeproduktion und -verlust zugrunde, die durch Aufenthalt in warmer Umgebung oder starke körperliche Aktivität ausgelöst wurde. Der Hitzschlag ist charakterisiert durch eine Körperkerntemperatur von > 40 °C, Krampfanfälle, Bewusstseinsstörungen und multiples Organversagen [46]. Unabhängig von der Sportart stellt der Belastungshitzschlag bei jungen Sportlern mit einer Mortalität von 21 bis 63 % die zweithäufigste Todesursache dar (Abb. 1; [16, 47, 48]). Entkleidung und dermale Kühlung (Eis, Tücher) sind Eckpunkte der präklinischen Therapie. Sicherung der Vitalfunktionen und symptomatische Therapie haben eine hohe Bedeutung. Bei hoher Körpertemperatur und vital bedrohlichen klinischen Symptomen muss eine zügige Einweisung ins Krankenhaus erfolgen.

##### Merke

Der Hitzschlag ist ein bedrohliches Krankheitsbild [46]. Bei Laufveranstaltungen muss bei allen Patienten die Körpertemperatur gemessen werden.

#### Nierenfunktion

Das Risiko, im Rahmen einer kurz- oder langstreckigen Laufveranstaltung eine Nierenfunktionseinschränkung zu entwickeln, ist beim gesamten Teilnehmerfeld erhöht [16]. Die Ursachen beinhalten

- eine katecholamininduzierte renale Vasokonstriktion mit resultierender Reduktion der glomerulären Filtrationsrate [16, 22],
- eine Einnahme von NSAR mit Einschränkung der renalen Durchblutung [16, 49],
- eine Reduktion des Plasmavolumens mit Aktivierung der Arginin-Vasopressin-Achse, die zu Flüssigkeitsretention und Oligurie führt [16, 22],
- das Einsetzen einer Rhabdomyolyse mit Freisetzung von Proteinen und Metaboliten [16, 22].

Die präklinische Diagnose der akuten Nierenfunktionsstörung ist aufgrund der heterogenen Klinik schwierig. Häufig zeigen sich symptomlose Verläufe. Weitere Manifestationen sind Kollaps, Elektrolytstörungen, Herzrhythmusstörungen, Oligurie, Polyurie, Anurie, metabolische Azidose, zentrale/periphere Ödeme und Muskelschwäche [16, 50]. Eine symptomatische Therapie, die Stabilisierung und eine zeitnahe Einweisung des Patienten sind wegweisend.

#### Gerinnungssystem

Langstreckenläufer unterliegen einem erhöhten Risiko für thromboembolische Ereignisse. Traumata der Gefäßwand, systemische Entzündungsreaktionen und Dehydratation verändern die Viskosität des Blutes. Belastungsinduzierte Bradykardie sowie Blutdruckanstiege und -abfälle verändern den Blutfluss [51]. Klinisch sind Verschlüsse der Lungenarterien [52] und der Herzkranzgefäße [53] sowie die Zentralvenenthrombose der Retina [54] nach einer Laufveranstaltung beschrieben. Im Rahmen des Hitzschlags wurde zudem von der Entwicklung einer disseminierten intravasalen Koagulopathie (DIC) berichtet [55].

#### Gastrointestinaltrakt

Das Auftreten von gastrointestinalen Symptomen bei Ausdauerläufern wird mit 30–90 % angegeben (Tab. 3; [56]). Die Schwere der Symptomatik korreliert mit der Distanz bzw. dem Ausmaß der körperlichen Belastung [56]. Ursächlich ist eine belastungsabhängige Sympathikusaktivierung mit Vasokonstriktion der Gefäße im Splanchnikusgebiet. Der intestinale Blutfluss wird konsekutiv um bis zu 80 % reduziert, was eine Ischämie der Darmmukosa begünstigt [56]. Bei hoher Belastung wird die gastrointestinale Motilität gehemmt und die intestinale Flüssigkeitsabsorption reduziert [57], was die Aspirationsgefahr bei Bewusstlosigkeit erhöht. Die Aufnahme von protein-, kohlenhydrat- und fettreicher Spezialnahrung fördert das Auftreten gastrointestinaler Symptome [56]. Leicht symptomatische Patienten zeigen Diarrhö, Defäkationsdrang, Erbrechen, Flatulenz, gastroösophagealen Reflux, Übelkeit, Krämpfe und Koliken. Ein Übergang zu schweren Symptomen wie abdomineller Angina, blutigen Durchfällen, Dünndarminfarzierung, hämorrhagischer Gastritis, ischämischer Kolitis und rektaler (okkulter) Blutung ist möglich [56, 57]. Die symptomatische Therapie und eine vorsichtige Rehydrierung sind präklinisch indiziert [56].

## Läuferspezifisches Risikoprofil

Sportliche Aktivität ist Bestandteil der Sekundärprophylaxe von Bluthochdruck, KHK und Diabetes mellitus. Amateurläufer zeigen häufig ein entsprechendes Risikoprofil, was bei der Patientenversorgung anamnestisch abgeklärt werden muss. Krankheitsbezogene Laufgruppen nehmen regelmäßig gemeinschaftlich an Laufveranstaltungen teil.

Ausdauersport kann bei Patienten mit Diabetes mellitus zu einer symptomatischen Hypoglykämie mit der Ausbildung einer Ketoazidose und neurologischer Symptomatik führen. Automatisierte Zuckersensoren und Insulinpumpen finden unter Sportlern breite Anwendung und können aufgrund von Fehlfunktionen oder fehlerhafter Bedienung zu rettungsdienstlichem Kontakt führen. Einige Laufveranstaltungen bieten parallel Wettkämpfe für Kinder und Schulklassen an, was das Einsatzspektrum erweitert.

### Merke

Alle vorbestehenden Erkrankungen können während körperlicher Anstrengung aggravieren.

### Substanzeinnahme

Die Einnahme ärztlich verordneter Substanzen muss von eigenständig durch den Läufer angesetzten Substanzen unterschieden werden. Der Übergang von Stoffen auf Grundlage einer medizinischen Indikation zur leistungssteigernden Substanz ist fließend.

#### Ärztlich verordnete Substanzen

Die Kombination von sportlicher Aktivität und der Einnahme von Statinpräparaten begünstigt das Auftreten einer Rhabdomyolyse [58]. Östrogene im Rahmen der Kontrazeption oder Osteoporosetherapie erhöhen das Risiko einer Thrombose [51]. Patienten mit bestehender antikoagulatorischer Medikation haben im Rahmen eines Traumas ein erhöhtes Risiko für Blutungskomplikationen.

#### Eigenständig angesetzte Substanzen

Küster et al. zeigten, dass 49 % der Marathonläufer vor dem Wettkampf NSAR, Cyclooxygenase(COX)-2-Inhibitoren oder Paracetamol zur Erhöhung der Schmerzschwelle einnehmen [49]. Insbesondere Einsteiger und Amateursportler mit nachlassender Leistungsfähigkeit gehören zur Risikogruppe [49]. Die Inhibition der Prostaglandinsynthese durch NSAR und COX-2-Inhibitoren kann zu Magengeschwüren, gastrointestinalen Blutungen, kardiovaskulären Ereignissen sowie Schädigung der Nierenfunktion führen [49, 59].

#### Leistungssteigernde Substanzen

Verschreibungspflichtige Substanzen ohne medizinische Indikation werden von 7,1 % aller Sportler bei Wettkampfveranstaltungen eingenommen [60]. Fehlende Kontrollen sowie schnelle und einfache Verfügbarkeit über digitale Bezugsquellen führen zu großer Verbreitung von leistungssteigernden Substanzen unter professionellen und Amateurathleten [61]. Das Spektrum leistungssteigernder Substanzen und ihrer Nebenwirkungen ist breit und nicht Gegenstand des vorliegenden Artikels. Medizinische Kontakte treten insbesondere im Rahmen der Einnahme von Stimulanzien auf, die über sympathoadrenerge Reaktionen eine zusätzliche Leistungssteigerung bewirken. Überschießende sympathomimetische Effekte begünstigen Tachykardien, Palpitationen, Herzrhythmusstörungen, Rhabdomyolyse und Myokardischämien [61, 62]. Langfristig angewandte anabole androgene Steroide (AAS) steigern das Risiko für Herzinsuffizienz und -infarkt sowie Leberschädigungen. Zudem werden AAS häufig in Kombination mit Insulin eingenommen und können zur Hypoglykämie führen [61].

#### Äußere Einflussfaktoren

Laufveranstaltungen gehen häufig mit Rahmenveranstaltungen einher. Die medizinische Versorgung einer hohen Anzahl an Zuschauern muss daher ebenfalls sichergestellt werden. Eine zusätzliche Risikobewertung der Rahmenveranstaltung mit dem von Aarau modifizierten Maurer-Schema stellt die Grundlage für die Bereitstellung ausreichender Ressourcen dar [63]. Durch umfangreiche Beeinträchtigung des Straßenverkehrs im Rahmen von Laufveranstaltungen kommt es zu verlängerten Anfahrtszeiten für Rettungsmittel [64]. Ein erhöhtes Patientenaufkommen verlängert die Warte- bzw. Versorgungszeit in Notaufnahmen für die gesamte Bevölkerung [64]. Für das Auftreten eines Massenanfalls von Verletzten (Terrorismus) muss die präklinische und klinische Versorgung einer großen Anzahl an Verletzten kurzfristig sichergestellt werden.

Oberhalb einer Lufttemperatur von 29 °C nehmen rettungsdienstliche Kontakte, Kollaps und Rennabbrüche zu [65–67]. Viele Autoren verwenden zur Beurteilung von Hitzestressbelastung auf den Menschen die sog. „wet bulb globe temperature“ (WBGT), die Effekte von Temperatur, Luftfeuchtigkeit, Windgeschwindigkeit und Sonnenstrahlung auf den Menschen berücksichtigt [68]. Die WBGT kann unkompliziert mittels Hitzestressmessgeräten gemessen werden. Roberts et al. empfehlen vor dem Hintergrund der Häufung medizinischer Zwischenfälle eine Absage der Laufveranstaltung ab einer WBGT von 21 °C [69].

##### Merke

Die Temperatur sollte während einer Laufveranstaltung mit adäquaten Methoden gemessen und kommuniziert werden. Der Rettungsdienst muss bei hohen Außentemperaturen auf entsprechende Erkrankungsmuster (z. B. Hitzschlag, Kollaps) vorbereitet sein.

## COVID-19

Auf Grundlage einer Computersimulation wird das Übertragungsrisiko für COVID-19 bei Laufveranstaltungen als sehr gering eingeschätzt. Starts in Kleingruppen reduzieren das Risiko weiter [70].

## Klinik und Diagnose

Unspezifische Symptome wie Erbrechen, Fieber, Kollaps, Muskelschmerzen, Übelkeit und Schwäche sind die führenden Symptome bei Laufveranstaltungen (Tab. 3). Wegweisend für die Erstversorgung und eine weiterführende klinische Therapie sind eine sorgfältige Anamnese und die vollständige Überprüfung der Vitalfunktionen. Einer „Bagatellisierung“ der Symptomatik durch den Läufer selbst sollte entgegengewirkt werden. Häufig kommt es nach dem Zieleinlauf innerhalb eines kurzen Zeitraums zu einem großen Patientenaufkommen, was bei der Planung der sanitätsdienstlichen Kräfte beachtet werden muss.

### Merke

Kernpunkte der präklinischen Versorgung von Patienten bei Laufveranstaltungen sind die genaue Anamnese sowie die gewissenhafte Überprüfung und Sicherung der Vitalfunktionen. Nach dem Zieleinlauf zeigen sich häufig erst Symptome von Erschöpfung.

## Fazit für die Praxis

- Der Kollaps infolge einer klassischen orthostatischen Dysregulation stellt die häufigste Ursache für medizinische Kontakte bei Langstreckenläufen dar.
- Unspezifische Symptome wie Erbrechen, Fieber, Kollaps, Muskelschmerzen, Übelkeit und Schwäche sind die führenden Symptome bei Laufveranstaltungen. Hauptaufgabe des Rettungsdiensts ist die Beurteilung der Schwere eines vorliegenden Krankheitsbilds und die rasche Zuführung in ein geeignetes Krankenhaus.
- Bewusstseinsstörungen und generalisierte Krampfanfälle sind schwerwiegende Komplikationen und können bei hohem Patientenaufkommen leicht übersehen werden.
- Der Kreislaufstillstand ist ein seltenes Ereignis bei Laufveranstaltungen.
- Die Hälfte der Marathonläufer nimmt nichtverschreibungspflichtige Substanzen zur Erhöhung der Schmerzschwelle ein.
- Das Flüssigkeitsmanagement stellt eine besondere Herausforderung unter präklinischen Bedingungen dar.

## Abkürzungen

AAMK: Anstrengungsassoziierter Muskelkrampf
AAS: Anabole androgene Steroide
COX: Cyclooxygenase
DIC: Disseminierte intravasale Koagulopathie (Gerinnung)
ILCOR: International Liaison Committee on Resuscitation
KHK: Koronare Herzkrankheit
NSAR: Nichtsteroidales Antirheumatikum
NSTEMI: Nicht-ST-Strecken-Elevations-Myokardinfarkt
STEMI: ST-Strecken-Elevations-Myokardinfarkt
